# Risk Scores in ST-Segment Elevation Myocardial Infarction Patients with Refractory Cardiogenic Shock and Veno-Arterial Extracorporeal Membrane Oxygenation

**DOI:** 10.3390/jcm10050956

**Published:** 2021-03-01

**Authors:** Carl Semaan, Arthur Charbonnier, Jeremy Pasco, Walid Darwiche, Christophe Saint Etienne, Xavier Bailleul, Thierry Bourguignon, Laurent Fauchier, Denis Angoulvant, Fabrice Ivanes, Thibaud Genet

**Affiliations:** 1 Service de Cardiologie, Centre Hospitalier Régional, Universitaire de Tours, 37044 Tours, France; c.semaan@chu-tours.fr (C.S.); arthurox380@hotmail.com (A.C.); w.darwiche@chu-tours.fr (W.D.); c.saintetienne@chu-tours.fr (C.S.E.); laurent.fauchier@univ-tours.fr (L.F.); denis.angoulvant@univ-tours.fr (D.A.); t.genet@chu-tours.fr (T.G.); 2 Faculté de Médecine, Université de Tours, 37032 Tours, France; thierry.bourguignon@univ-tours.fr; 3 Service d’Informatique Médicale, Épidémiologie et Économie de la Santé, Centre Hospitalier Régional, Universitaire de Tours, 37044 Tours, France; jeremypasco37@gmail.com; 4 Service de Chirurgie Cardiaque, Centre Hospitalier Régional, Universitaire de Tours, 37044 Tours, France; x.bailleul@chu-tours.fr

**Keywords:** extracorporeal membrane oxygenation, cardiogenic shock, acute myocardial infarction, outcome assessment

## Abstract

Although many risk models have been tested in patients implanted by veno-arterial extracorporeal membrane oxygenation (VA-ECMO), few scores assessed patients’ prognosis in the setting of ST-segment elevation myocardial infarction (STEMI) with refractory cardiogenic shock. We aimed at assessing the performance of risk scores, notably the prEdictioN of Cardiogenic shock OUtcome foR AMI patients salvaGed by VA-ECMO (ENCOURAGE) score, for predicting mortality in this particular population. This retrospective observational study included patients admitted to Tours University Hospital for STEMI with cardiogenic shock and requiring hemodynamic support by VA-ECMO. Among the fifty-one patients, the 30-day and 6-month survival rates were 63% and 56% respectively. Thirty days after VA-ECMO therapy, probabilities of mortality were 12, 17, 33, 66, 80% according to the ENCOURAGE score classes 0–12, 13–18, 19–22, 23–27, and ≥28, respectively. The ENCOURAGE score (AUC of the Receiving Operating Characteristic curve = 0.83) was significantly better compared to other risk scores. The hazard ratio for survival at 30 days for each point of the ENCOURAGE score was 1.10 (CI 95% (1.06, 1.15); *p* < 0.001). Decision curve analysis indicated that the ENCOURAGE score had the best clinical usefulness of the tested risk scores and the Hosmer–Lemeshow test suggested an accurate calibration. Our data suggest that the ENCOURAGE score is valid and the most relevant score to predict 30-day mortality after VA-ECMO therapy in STEMI patients with refractory cardiogenic shock. It may help decision-making teams to better select STEMI patients with shock for VA-ECMO therapy.

## 1. Introduction

The Veno-arterial “ExtraCorporeal Membrane Oxygenation” (VA-ECMO) is a temporary cardiopulmonary support that plays an increasingly important role in the management strategy of acute myocardial infarction (AMI) complicated with refractory cardiogenic shock [1,2,3,4,5,6,7]. Despite the progress of early revascularization, in particular with primary angioplasty (PA), AMI complicated by cardiogenic shock is associated with a mortality approximating 50% at one year [8,9,10]. VA-ECMO may be used to stabilize the patient with refractory cardiogenic shock post-AMI while awaiting possible myocardial recovery [11]. The decision to implant VA-ECMO in the acute phase of refractory cardiogenic shock secondary to AMI should be made as soon as possible to avoid implantation at the stage of multi-organ failure, which is associated with increased mortality [12,13]. Many clinical and paraclinical parameters, as well as the team’s expertise, all play a role in the VA-ECMO management strategy. This strategy also includes an ethical reflection on the patient and his prognosis, along with the matter of resources in terms of caregivers, infrastructure and costs.

In order to assist physicians in the selection of patients for the implantation of a VA-ECMO, the literature identified many prognostic factors, and several prognostic scores are now available [12,14,15]. These prognostic factors and scores cannot solely replace a complex and multidisciplinary decision; however, they constitute interesting tools for an appropriate selection of patients. The SAVE score (Survival After Veno-arterial ECMO) (available online: http://www.save-score.com (accessed on 1 December 2020)) is currently the most robust [14]. However, it is not specific to AMI-related refractory cardiogenic shock and applies to all indications for implantation of a VA-ECMO. Yet, AMI complicated by refractory cardiogenic shock is the most frequent indication for implantation of VA-ECMO. The recent ENCOURAGE score (“prEdictioN of Cardiogenic shock OUtcome foR AMI patients salvaGed by VA-ECMO”) enabled to identify several pre-ECMO factors associated with 30-day mortality in AMI patients complicated by refractory cardiogenic shock and implanted by a VA-ECMO [16]. This score was established based on a retrospective cohort of 138 patients from two French intensive care units and aimed at improving patients’ selection for the implantation of a VA-ECMO. External validation was recently sought, but the characteristics of the validation cohort were different from the ENCOURAGE population regarding numerous parameters, with a mix of ST- and non-ST-segment elevation AMI and only 49% PA [17].

The main objectives of our study were to further validate the ENCOURAGE prognostic score in an independent cohort of patients with ST-segment elevation myocardial infarction (STEMI) complicated by refractory cardiogenic shock and compare its performance to other mortality risk scores commonly used in resuscitation that may be used in this setting.

## 2. Materials and Methods

### 2.1. Patients

All consecutive patients who benefited from a VA-ECMO implanted at Tours University Hospital between March 2007 and March 2017 for STEMI-related refractory cardiogenic shock were included in this work. This retrospective monocentric observational study was approved by the institutional review board of the Pole Coeur Thorax Vaisseaux from the Trousseau Hospital (Tours, France) on December 2017 and was registered as a clinical audit of routine care. Participating patients’, or their families’, non-opposition was recorded.

Patients with refractory cardiogenic shock following an AMI eligible for VA-ECMO usually correspond to the Interagency Registry for Mechanically Assisted Circulatory Support (INTERMACS) profiles 1 and 2. INTERMACS 1 refers to a cardiogenic shock in critical state (life-threatening arterial hypotension, rapid escalation of the vasopressor support, severe organ hypoperfusion with lactic acidosis). INTERMACS 2 refers to a cardiogenic shock gradually deteriorating (dependence on intravenous catecholamines, deterioration of the renal/hepatic function, ongoing congestive signs). When a patient fulfilled these criteria for circulatory support, the indication was immediately confirmed by a multidisciplinary team comprising at least one cardiothoracic surgeon, a physician from the intensive care unit, and an interventional cardiologist. The VA-ECMO were inserted either through surgical approach in the operating room by a cardiothoracic surgeon, or more frequently using a percutaneous approach in the Cath Lab by a trained interventional cardiologist. After VA-ECMO therapy, all patients were monitored in the Cardiothoracic and Vascular Surgery Resuscitation Department of Tours University Hospital.

Exclusion criteria were as follows: life expectancy <1 year, prolonged cardiac arrest before VA-ECMO therapy (>60 min), iatrogenic myocardial infarction secondary to a percutaneous coronary intervention, and VA-ECMO implanted in another center before admission in Tours University Hospital.

### 2.2. Data Collection and Score Calculation

Clinical and paraclinical data were collected afterwards thanks to hospital reports, additional examination reports, biological results, and the patients’ Electronic Health Records of Tours University Hospital. To assess survival, we sought for the date of the STEMI and the date of implantation of the VA-ECMO, the date on which the patient left the intensive care unit, the last date of follow-up for living patients, and the date of death, if applicable. ENCOURAGE, SAVE (Survival after Veno-Arterial ECMO) and SOFA (Sequential Organ Failure Assessment) scores were calculated with parameters available at the date of implantation of the VA-ECMO so that these scores could be compared.

We also collected the following data: cardiovascular risk factors (smoking habits, dyslipidemia, arterial hypertension, diabetes mellitus, family history of coronary heart disease); vascular history (peripheral artery disease, history of Coronary Artery Bypass Graft (CABG) or coronary angioplasty); data regarding the STEMI (localization, initial intravenous fibrinolysis, PA); data in the event of a cardiac arrest (duration, presence of a shockable rhythm, implantation of a VA-ECMO under cardiopulmonary resuscitation); data concerning the VA-ECMO (surgical or percutaneous implantation, association with an Intra-Aortic Balloon Pump (IABP), number of days under VA-ECMO); data during the stay in intensive care unit (SAPS (Simplified Acute Physiology Score) II score, number of days under mechanical ventilation, renal replacement therapy); and complications of VA-ECMO (severe bleeding according to the Bleeding Academy Research Consensus, acute limb ischemia, scarpa infection, pulmonary edema, hemolysis). Patients’ outcome was classified into 4 categories: death, VA-ECMO weaning, heart transplant, or LVAD implantation.

The ENCOURAGE score was calculated for each patient as described in the article from Muller et al. based on data available prior to VA-ECMO therapy (16). The score calculation is available in Table S1 (Supplementary Materials).

The patients were classified as survivors or non-survivors and divided into 5 groups corresponding to the 5 classes of the ENCOURAGE score: 0–12, 13–18, 19–22, 23–27, and ≥28.

### 2.3. Statistical Analysis

Depending on their distribution, continuous variables were expressed as mean (± standard deviation) or median (25th and 75th percentiles). The comparison between the survivors and non-survivor groups was carried out either using a two-tailed Student t-test or using a Mann–Whitney test depending on whether the distribution was normal or not. Categorical variables were expressed as percentages and Chi-square tests were used for comparison. Kaplan–Meier survival curves were drawn for each group of ENCOURAGE score in order to estimate the probability of survival after VA-ECMO initiation and compared with the log-rank test. The Hazard Ratio and the Odds Ratio were calculated for each point of the ENCOURAGE score. The area under the ROC curve (AUC) was used to study the predictive performance the different scores in terms of mortality and compare them to the ENCOURAGE score. The ROC curves were then compared using the DeLong test. Decision-curve analysis (DCA) was used to quantify the clinical usefulness of the prediction models. Model calibration was considered acceptable at the Hosmer–Lemeshow goodness-of-fit test *p* > 0.1. We then plotted observed versus predicted risks by decile of predicted risk, and the regression line was compared against the line of equality (intercept = 0, slope = 1). All statistical analyses were performed using the R software version 3.3.2 and the JMP 9 software (version 9.0.1, SAS institute, Cary, NC, USA) and the Stata/SE (version 16.0, StataCorp, College Station, TX, USA) [18]. A value of *p* < 0.05 was considered statistically significant.

## 3. Results

### 3.1. Study Population

One hundred and six patients were implanted with VA-ECMO for cardiogenic shock in our Intensive Care Unit between March 2007 and March 2017, the flow chart is described in Figure S1 (Supplementary Materials). Fifty-five patients were excluded. Fifty-one patients implanted with VA-ECMO for STEMI-related refractory cardiogenic shock were included in our cohort.

The population characteristics are described in Table 1. The median age was 53 (47–58) years and there was a male prevalence (M/F ratio 4/1). A total of 76% of patients had anterior STEMI and successful revascularization with PA was achieved in 87%.

Our cohort was comparable to the ENCOURAGE cohort [16] in terms of age, with, respectively, 53 years (47–58) and 55 years (46–63), and gender, with 80% of men in both populations. All patients had ST-segment elevation AMI with a similar percentage of anterior localization (76% versus 67%, respectively), 12% of our patients were implanted with VA-ECMO under CPR (14% of the ENCOURAGE patients) but arterial lactates levels were lower (3.1 versus 4.1 mmol/L).

### 3.2. Survival Results and Survival-Related Variables

Thirty-two patients (63%) were alive 30 days after the implantation of the VA-ECMO. All left the intensive care unit alive. The median duration of VA-ECMO hemodynamic support was 7 days, while the median hospital stay was 27 days. The outcomes and complications that occurred during the VA-ECMO period are presented in Table 2. Among the 51 patients of our cohort, 17 (33%) were successfully weaned from VA-ECMO with medical treatment only; 11 (22%) received a heart transplant; and 7 (13%) underwent LVAD implantation. Among these seven patients, two secondarily received a heart transplant. At 6 months, 29 patients (56%) were still alive. Figure S2 (Supplementary Materials) displays the flow diagram.

### 3.3. Performance of the ENCOURAGE Score in Predicting the 30-Day Survival

**Figure 1:**
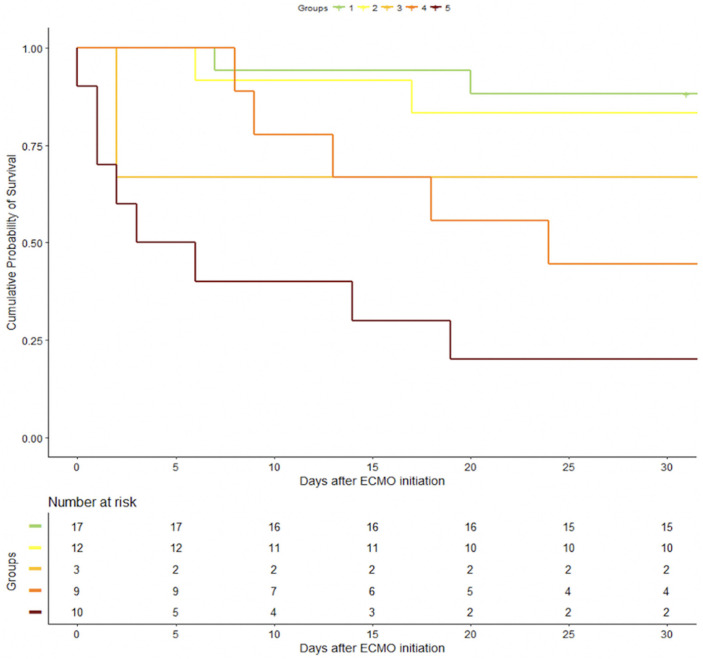
Kaplan–Meier curves estimating the 30-day survival of patients implanted with a VA- veno-arterial extracorporeal membrane oxygenation (ECMO) for STEMI-related refractory cardiogenic shock.

Figure 1 represents the Kaplan–Meier curves estimating the 30-day survival in our study. The probability of mortality at 30 days increases for each score class with mortality rates of 12, 17, 33, 66, and 80% for classes 0–12, 13–18, 19–22, 23–27, and ≥28, respectively (*p* < 0.001 for log-rank test).

Table S2 (Supplementary Materials) shows the expected survival according to the ENCOURAGE score and the observed survival in our cohort.

The Hazard Ratio for the 30-day mortality per point of the ENCOURAGE score in our cohort was 1.10 with a 95% CI (1.06; 1.15) and a *p* < 0.001.

### 3.4. Comparison of the ENCOURAGE Score with Other Scores

**Figure 2:**
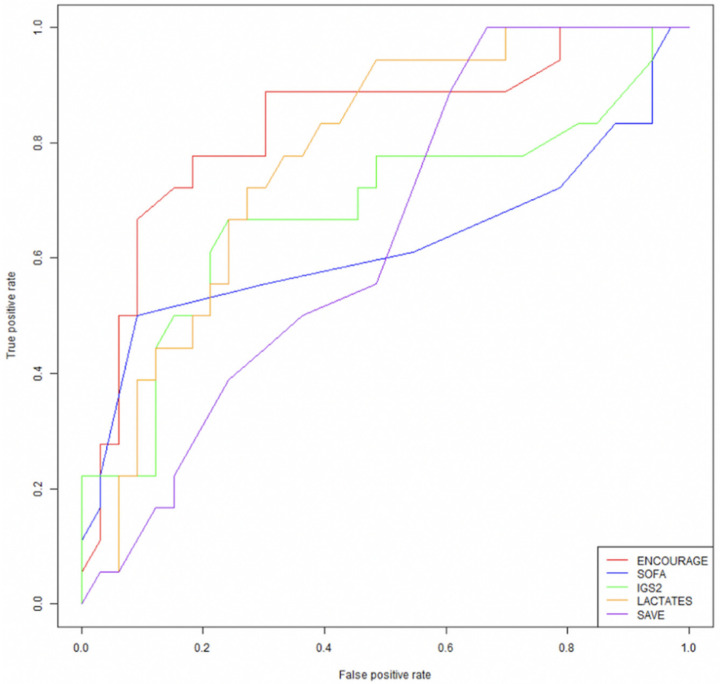
Receiving Operating Characteristic (ROC) curve of the prEdictioN of Cardiogenic shock OUtcome foR AMI patients salvaGed by VA-ECMO (ENCOURAGE), Sequential Organ Failure Assessment (SOFA), Simplified Acute Physiology Score II/Indice de Gravité Simplifié (SAPS II/IGS2), lactate, and Survival After Veno-arterial ECMO (SAVE) scores to predict the 30-day mortality.

The performance of the ENCOURAGE, SAPS II [19], SOFA [20], and SAVE [14] scores to predict 30-day mortality is represented in Figure 2. The ENCOURAGE score had the best area under the ROC curve (AUC) with 0.86 (with a 95% CI 0.74–0.97) as compared to 0.81 (0.69–0.93) for arterial lactate levels (*p* = 0.28 for DeLong test versus ENCOURAGE), 0.71 (0.55–0.87) for the SAPS II score (*p* = 0.11 for DeLong test versus ENCOURAGE), 0.67 (0.52–0.82) for the SAVE score (*p* = 0.01 for DeLong test versus ENCOURAGE), and 0.66 (0.47–0.84) for the SOFA score (*p* = 0.05 for DeLong test versus ENCOURAGE).

**Figure 3:**
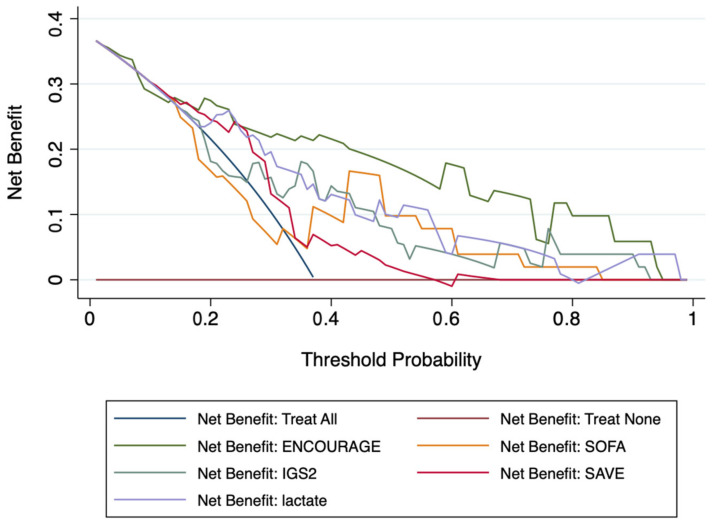
Decision curve analysis: net number of true positives gained using different models compared to no model at a range of thresholds probabilities.

Decision curve analysis indicated that the ENCOURAGE score had the best clinical usefulness of the tested risk scores for the 30-day mortality (Figure 3).

Regarding calibration of the ENCOURAGE Score, the Hosmer–Lemeshow goodness of fit test had a *p*-value of 0.70 suggesting that the model was accurate. The observed versus predicted risks of all-cause death at day 30 within risk quintiles for each score are shown in Supplemental Figure S3. Calibration of the ENCOURAGE score was satisfying across the several plots and a predicted rate of death at day 30 from 0 to 100%, whilst this was far less clearly seen with other prediction tools.

## 4. Discussion

Our single center retrospective study allows validation of the use of the ENCOURAGE score as the best predictive score of the 30-day mortality currently available in patients implanted by VA-ECMO for STEMI-related refractory cardiogenic shock [16]. This is the first score developed solely on parameters preceding the use of VA-ECMO and in a population consisting of only ST-segment elevation myocardial infarction patients, which corresponds to our study population.

In our study, the 30-day mortality rate was 37%, which is comparable with the cohort from Pabst et al. (41%) [17] but lower compared to the ENCOURAGE cohort (53%), and, on a larger scale, to the mortality observed in patients with AMI complicated with cardiogenic shock, whether implanted with VA-ECMO [21] or not [9,22]. Such differences may be explained by the fact that our patients presented fewer criteria of severity at the time of VA-ECMO therapy compared to the ENCOURAGE patients. Seventeen of our patients had an ENCOURAGE score between 0 and 12 and the median lactate level in our population was 3.1 mmol/L compared to 4.1 in the ENCOURAGE study. In addition, the rate of cardiac arrest was much lower in our cohort (33% compared to 57% in the ENCOURAGE cohort), though only few patients in both cases were implanted under CPR. The occurrence of cardiac arrest is a factor associated with poor prognosis, in particular if the patient is still undergoing cardiopulmonary resuscitation during VA-ECMO therapy, whatever the etiology of the cardiac arrest, according to Combes et al., in a study that included 16 AMI patients out of 81 [13]. Yet, there are reports advocating an absence of increased mortality in AMI patients when cardiac arrest recovered during the acute phase [23]. Additionally, the incidence of complications with VA-ECMO in our study was comparable to data previously published and does not explain this difference in survival [24]. The success rate of VA-ECMO weaning was 33% in our study, similar to that of the ENCOURAGE study (36%). Notably, in the cohort from Pabst et al., 61% of patients were weaned from VA-ECMO; yet this population had a different profile from ours, with more than one-third non-ST-segment elevation AMI, 30% of patients with a left ventricular ejection fraction above 35%, 49.2% were treated with PA, 36.1% with coronary artery bypass grafts and up to 14.8% without revascularization. For comparison, in our study, we included only STEMI patients, mainly anterior STEMI, complicated by refractory cardiogenic shock, with 92% of PA, a much more homogeneous population managed in accordance with current European and American guidelines on the management of STEMI.

The difference in survival rates between our cohort and the ENCOURAGE cohort may therefore be attributed to the management of patients with failed VA-ECMO weaning. In the absence of myocardial recovery and the impossibility of weaning from VA-ECMO, the therapeutic options are heart transplantation or the implantation of a LVAD or BiVAD [25,26]. In our study, 22% of patients underwent heart transplantation compared to 9% in the ENCOURAGE cohort. All transplanted patients were alive at 30 days. LVAD implantation rates were similar in both studies. The issue highlighted here is that myocardial recovery remains unpredictable. It could be interesting to identify as early as possible the patients who may be secondarily eligible for a LVAD or heart transplantation in case of the absence of recovery. However, such selection appears not feasible in routine clinical practice since VA-ECMO therapy is most often done urgently as a “bridge to decision” [26]. Yet, it seems essential, from the initial management of these patients, to assess in a simple way whether there may be any obstacle to LVAD implantation or heart transplantation. The increase in pulmonary vascular resistance, which is considered as a contraindication to heart transplantation, is often absent in these patients who most often did not suffer from any heart disease before the AMI [27]. Increased age, high BMI, and renal failure are parts of the ENCOURAGE score but will also hinder any possible heart transplantation in the absence of recovery. The prognostic impact of these factors is therefore two-fold. For the LVAD, the essential parameter conditioning success is the right ventricular function. Yet, once the ECMO is placed, the drainage of the right ventricle will make its assessment difficult. It is therefore important to assess this right ventricular function before placing the ECMO to find out whether LVAD-type assistance may be possible in the course of “destination therapy” or “bridge to candidacy” [28]. In the absence of an available graft, when there is a right ventricular dysfunction, the implantation of a BiVAD may be considered [26]. The current shortage of grafts tends to increase the number of complex situations and the need to consider LVAD or BiVAD-type assistance [29]. In the absence of myocardial recovery, the reasonable waiting time for a heart transplant on VA-ECMO is not clearly established. A recent study highlighted the benefit of an early bridge-to-bridge, which was associated with better survival rates [30]. Of course, in countries where these therapeutic options are not available or are more difficult to access than in France, the survival of these patients may significantly differ. Yet this should not impact the ability of the ENCOURAGE score to identify the patients with the best/worst prognosis.

Among the different types of temporary assistance, VA-ECMO is currently the most used option with an increasing number of implantations [31]. This is due in particular to its relative simplicity of implantation and its reasonable cost. To date, however, there has been no randomized study of VA-ECMO in AMI-related cardiogenic shock [25,26]. A recent meta-analysis reported that the use of VA-ECMO in patients with refractory cardiac arrest and AMI-related cardiogenic shock significantly improved survival rates [32], which was not the case for the IABP. Data regarding the efficacy of the Impella temporary left ventricular assistance remain contradictory, though a recent article by Karami et al. supports the usefulness of the high-output Impella [33]. Yet, if the efficacy of VA-ECMO in AMI complicated with cardiogenic shock remains to be demonstrated with larger randomized studies, there is no doubt of the benefit of timely revascularization, even in this critically ill population [34,35]. Notably, the implantation of IABP along with the ECMO is almost systematic in our cohort. IABP in this setting allows left ventricle discharge, which may promote its recovery and prevent the occurrence of pulmonary edema [36,37]. This is supported by a study of Aso et al. that showed better survival rates for patients on VA-ECMO when it was associated with an IABP [38].

The ENCOURAGE score was derived from a population of patients with AMI-related cardiogenic shock all implanted by VA-ECMO. Its use to better select patients eligible for a VA-ECMO is therefore an extrapolation that requires evaluation. This score could in fact be used to layer the patients’ risk level in a randomized study evaluating VA-ECMO in patients with an AMI-related refractory cardiogenic shock. Several parameters constituting the ENCOURAGE score were described in the literature as factors of poor prognosis in AMI-related cardiogenic shock, independently of the implantation of a VA-ECMO. They include increased age [10,39], female gender [40], and increased serum creatinine [8,40]. Additionally, increased age [2,14,41], female gender [13], increased BMI [42], impaired renal function [13,14,43], impaired liver function [13], and neurological disorders [14] are associated with poor prognosis in patients implanted with VA-ECMO for cardiogenic shock, whatever the etiology of the shock. Notably, increased age also negatively impacts lengths of stay and costs [41]. A recent study considered lactate clearance at the initiation of ECMO as a significant predictor of 30-day mortality along with the delay in revascularization [42]. Revascularization failure is well described in the literature as a factor of poor prognosis [2,43], but this parameter did not emerge in the ENCOURAGE cohort where the success rate of revascularization was 88% (87% in our study).

Our study has however several limitations that characterize most studies in this setting, i.e., the fact that it was retrospective, non-randomized, from a single center. Our population was smaller than the population of the original cohort from Muller et al., which was a two-center study, and from the population of the validation study from Pabst et al., though they had less STEMI patients. In our study, we included only STEMI patients, mainly anterior STEMI, complicated by refractory cardiogenic shock. The physiopathology of cardiogenic shock is somewhat different between STEMI and non-STEMI patients, where the non-STEMI plays mainly the role of trigger in a situation where the myocardium is already ill. In the context of STEMI, the multiple organ failure originates from systemic inflammation secondary to the STEMI and is associated with vasoplegia, which, in addition to the decrease in cardiac output secondary to the myocardial ischemia, leads to inotropic support requirement [44]. Notably, the ENCOURAGE seems to have poor calibration for patients with 19–22 points. This may simply be due to the small number of patients (only three) in this group. Yet, it provides new data regarding the relevance of risks scores in STEMI-related refractory cardiogenic shock requiring VA-ECMO.

## 5. Conclusions

Our study validates the ENCOURAGE score as the best risk score to assist physicians in predicting the 30-day mortality of STEMI patients complicated with refractory cardiogenic shock and implanted by VA-ECMO. This simple prognostic score can be easily used in clinical practice. However, this score should in no case supersede multidisciplinary consultation. The latter should incorporate the characteristics of the patient before the occurrence of STEMI, at the time of the infarction, and at the time of refractory cardiogenic shock (including the occurrence of cardiac arrest), but also parameters that may quickly qualify the patient for another therapeutic option, i.e., LVAD or heart transplantation, in the absence of myocardial recovery.

## Figures and Tables

**Table 1:** Patient characteristics according to the Intensive Care Unit (ICU) survival status.

Variables	All Patients (*n* = 51)	Survivors (*n* = 32)	Non-Survivors (*n* = 19)	*p*-Value
Age (years)	53 (47–58)	52 (42–57)	54 (51–64)	0.14
Male Gender	41 (80)	27 (84)	14 (73)	0.35
BMI (kg/m^2^)	26 (23–28)	25 (22–28)	27 (25–29)	0.17
CV Risk Factors				
Smoking habits	35 (69)	22 (69)	13 (68)	0.98
Dyslipidemia	12 (23)	7 (21)	5 (26)	0.72
Arterial Hypertension	21 (41)	15 (47)	6 (32)	0.28
Diabetes mellitus	11 (22)	7 (22)	4 (21)	0.94
Family history of CHD	6 (12)	2 (6)	4 (21)	0.11
Vascular History				
Previous coronary angioplasty or CABG	3 (6)	1 (3)	2 (11)	0.28
Peripheral artery disease	3 (6)	1 (3)	2 (11)	0.28
SOFA Score	9 (8–10)	9 (8–10)	11 (7–12)	0.09
SAPS II Score	38 (29–44)	33 (29–40)	44 (35–67)	0.02
STEMI-ECMO Period (days)	1 (0–4)	1 (0–7)	0 (0–3)	0.36
Pre-ECMO Cardiac Arrest	17 (33)	9 (28)	8 (42)	0.31
No Flow duration (min)	0 (0–0)	0 (0–0)	0 (0–10)	
Low Flow duration (min)	20 (12–45)	10 (12–20)	30 (20–40)	
Shockable Rhythm	12/17 (71)	8/9 (89)	4/8 (50)	
ECMO under CPR	6 (12)	1 (3)	5 (26)	0.01
Intravenous fibrinolysis	3 (6)	3 (10)	0	0.17
Anterior STEMI	39 (76)	24 (75)	15 (79)	0.75
Primary Angioplasty	47 (92)	29 (90)	18 (95)	0.60
Successful PA	41/47 (87)	26/29 (90)	15/18 (83)	
Post-PA ECMO	50 (98)	31 (96)	19 (100)	0.43
Time from PA to ECMO (h)	7 (0–84)	8 (0–114)	2 (0–52)	0.49
Percutaneous ECMO implantation	23 (45)	13 (57)	10 (43)	0.56
Distal perfusion cannulation	51 (100)	32 (100)	19 (100)	
ECMO-related IABP	46 (90)	30 (94)	16 (84)	0.27
Pre-ECMO IABP	27 (53)	20 (62)	7 (37)	0.08
Pre-ECMO MV	51 (100)	32 (100)	19 (100)	
Glasgow Coma Scale score	14 (3–14)	14 (14–14)	3 (3–14)	0.03
Arterial Lactate (mmol/L)	3.1 (1.9–7.3)	2.2 (1.5–3.7)	6.5 (3.2–13.3)	<0.01
Prothrombin ratio (%)	55 (45–66)	55 (53–70)	45 (36–55)	<0.01
Serum Creatinine (µmol/L)	122 (100–170)	115 (93–176)	149 (115–167)	0.52
Arterial pH	7.32 (7.29–7.43)	7.32 (7.31–7.43)	7.32 (7.19–7.44)	0.08

Values expressed as median (25th and 75th percentiles) or as *n* (%) unless specified, *p*-values are provided for comparisons between survivors and non-survivors. STEMI, ST-segment Elevation Myocardial Infarction; BMI, Body Mass Index; CABG, Coronary Artery Bypass Graft; CHD, Coronary Heart Disease; CPR, Cardiopulmonary Resuscitation; CV, Cardiovascular; ECMO, extracorporeal membrane oxygenation; FMC, First Medical Contact; IABP, Intra-aortic balloon pump; MV, Mechanical Ventilation; PA, Primary Angioplasty; SAPS II, Simplified Acute Physiology Score II; SOFA, Sequential Organ Failure Assessment

**Table 2:** Hospital survival-related variables.

Variables	All Patients (*n* = 51)	Survivors (*n* = 32)	Non-Survivors (*n* = 19)	*p*-Value
ECMO Duration (days)	7 (4–14)	7 (5–12)	6 (2–15)	0.54
Hospital Stay (days)	27 (12–40)	34 (28–54)	7 (2–17)	<0.01
Weaned off ECMO/recovery	17 (33)	16 (50)	1 (5)	<0.01
LVAD Assist	7 (13)	5 (16)	2 (11)	0.61
Heart transplant	11 (22)	11 (34)	0	<0.01
BARC III bleeding event	17 (33)	8 (25)	9 (47)	0.10
Acute Limb Ischemia	8 (16)	3 (9)	5 (26)	0.11
Scarpa Infection	5 (10)	4 (13)	1 (5)	0.40
Acute Pulmonary Edema	16 (31)	7 (22)	9 (47)	0.06
Renal replacement therapy	12 (23)	4 (12)	8 (42)	0.01
Left ventricle thrombus	6 (12)	0 (0)	6 (32)	<0.01
Harlequin syndrome	4 (8)	0 (0)	4 (21)	0.02
Switch from peripheral to Central ECMO	2 (4)	0	2 (10)	0.04
Massive Hemolysis	0	0	0	

Values expressed as median (25th and 75th percentiles) or as *n* (%), *p*-values are provided for comparisons between survivors and non-survivors. LVAD, Left Ventricular Assist Device; BARC, Bleeding Academy Research Consensus.

## Data Availability

The datasets used and/or analyzed during the current study are available from the corresponding author on reasonable request.

## Supplementary Materials

The following are available online at https://www.mdpi.com/2077-0383/10/5/956/s1, Figure S1: Flow chart; Figure S2: Diagram representing 30-day and 6-month survival; Figure S3: Calibration plots of the scores predicting the 30-day mortality; Table S1: ENCOURAGE score calculation, independent pre-VA-ECMO variables associated with mortality; Table S2: expected survival at 30 days and observed survival at 30 days according to the 5 classes of the ENCOURAGE Score.
