# Supplementary material for: Risk Scores in ST-Segment Elevation Myocardial Infarction Patients with Refractory Cardiogenic Shock and Veno-Arterial Extracorporeal Membrane Oxygenation
Source: J Clin Med. 2021 Mar 1;10(5):956. doi: 10.3390/jcm10050956 (PMC7957612; doi:10.3390/jcm10050956)

Journal of
Clinical Medicine

MDPI

Original research article

Risk Scores in ST-Segment Elevation Myocardial Infarction Patients with Refractory Cardiogenic Shock and Veno-Arterial Extracorporeal Membrane Oxygenation

Carl Semaan et al.

SUPPLEMENTARY MATERIAL

Table S1: ENCOURAGE Score Calculation, Independent pre-VA-ECMO variables associated with mortality

Variables	ENCOURAGE Score: Number of Points
Age > 60 years	5
Female Gender	7
Body Mass Index > 25 kg/m^2	6
Glasgow Coma Scale Score < 6	6
Serum Creatinine > 150 µmol/l	5
Arterial Lactate	
< 2 mmol/l	0
2-8 mmol/l	8
> 8 mmol/l	11
Prothrombin ratio < 50 %	5

According to Muller *et al.* 2016

Table S2: Expected Survival at 30 Days and Observed Survival at 30 days According to the 5 Classes of the ENCOURAGE Score.

ENCOURAGE Score Classes	ENCOURAGE Score Expected Survival	Observed Survival in our Cohort (n=51)
0 – 12 points	92 %	88 %
13 – 18 points	70 %	83 %
19 – 22 points	35 %	67 %
23 – 27 points	28 %	44 %
≥ 28 points	17 %	20 %

According to Muller *et al.* 2016

Figure S1: Flow Chart

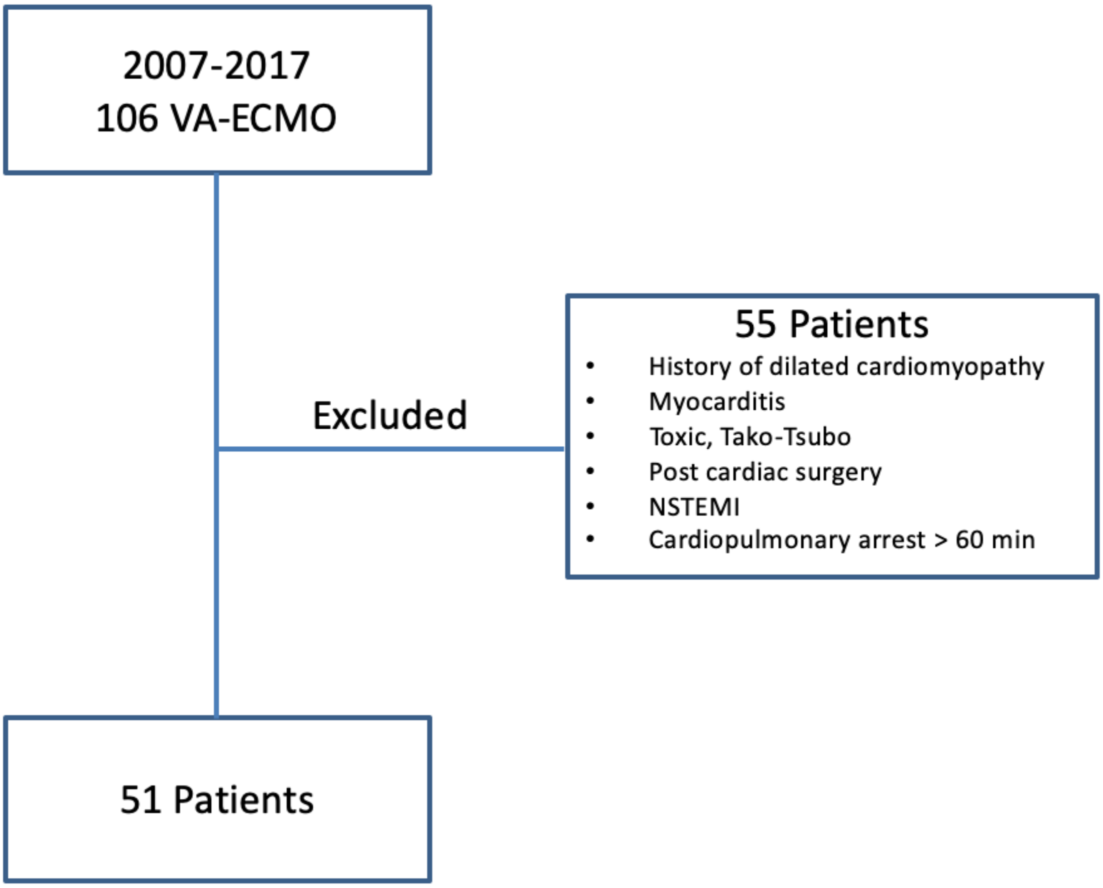

Figure S2: Diagram representing 30-day and 6-month survival

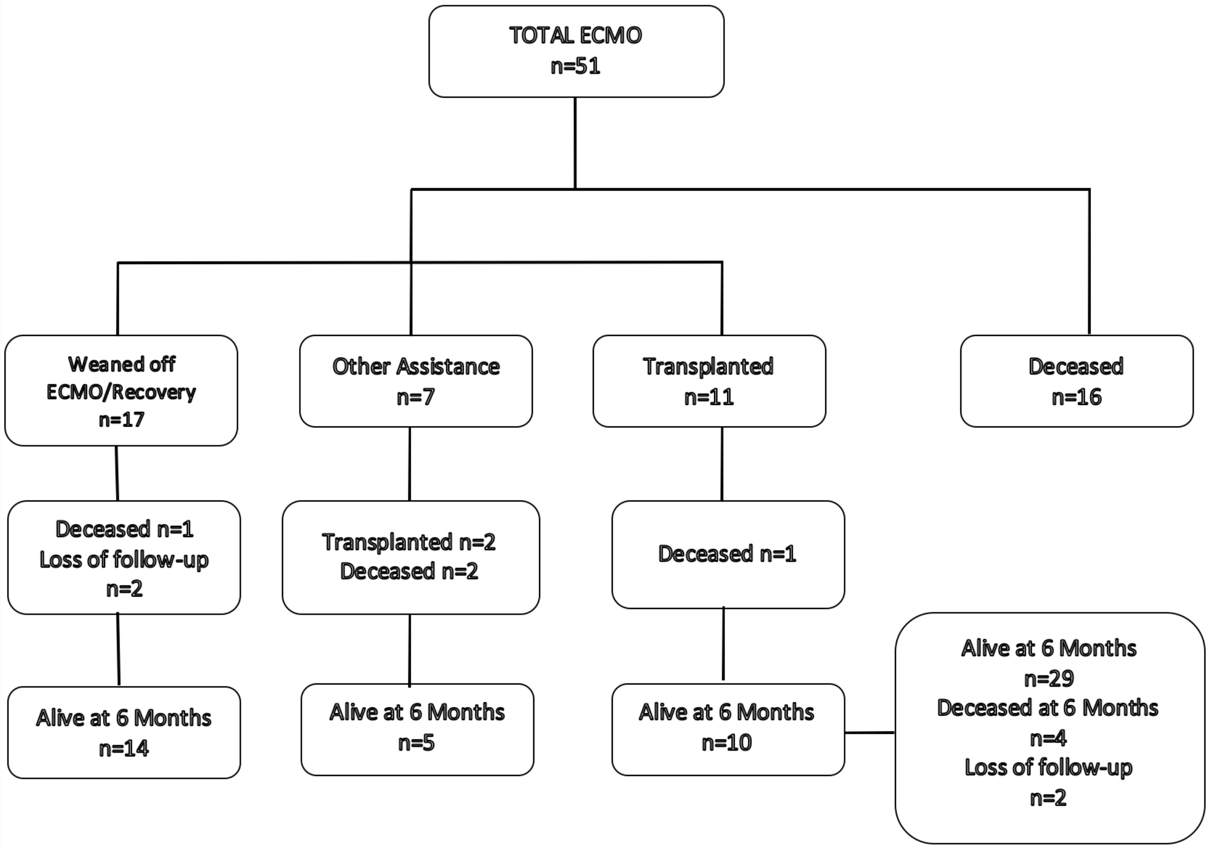

Figure S3: Calibration plots of the scores predicting the 30-day mortality with ENCOURAGE score (top panel), SOFA score (middle left panel), SAPS II score (middle right panel), lactate (lower left panel) or SAVE score (lower right panel). The diagonal line represents perfect calibration. Vertical bars represent 95% CIs.

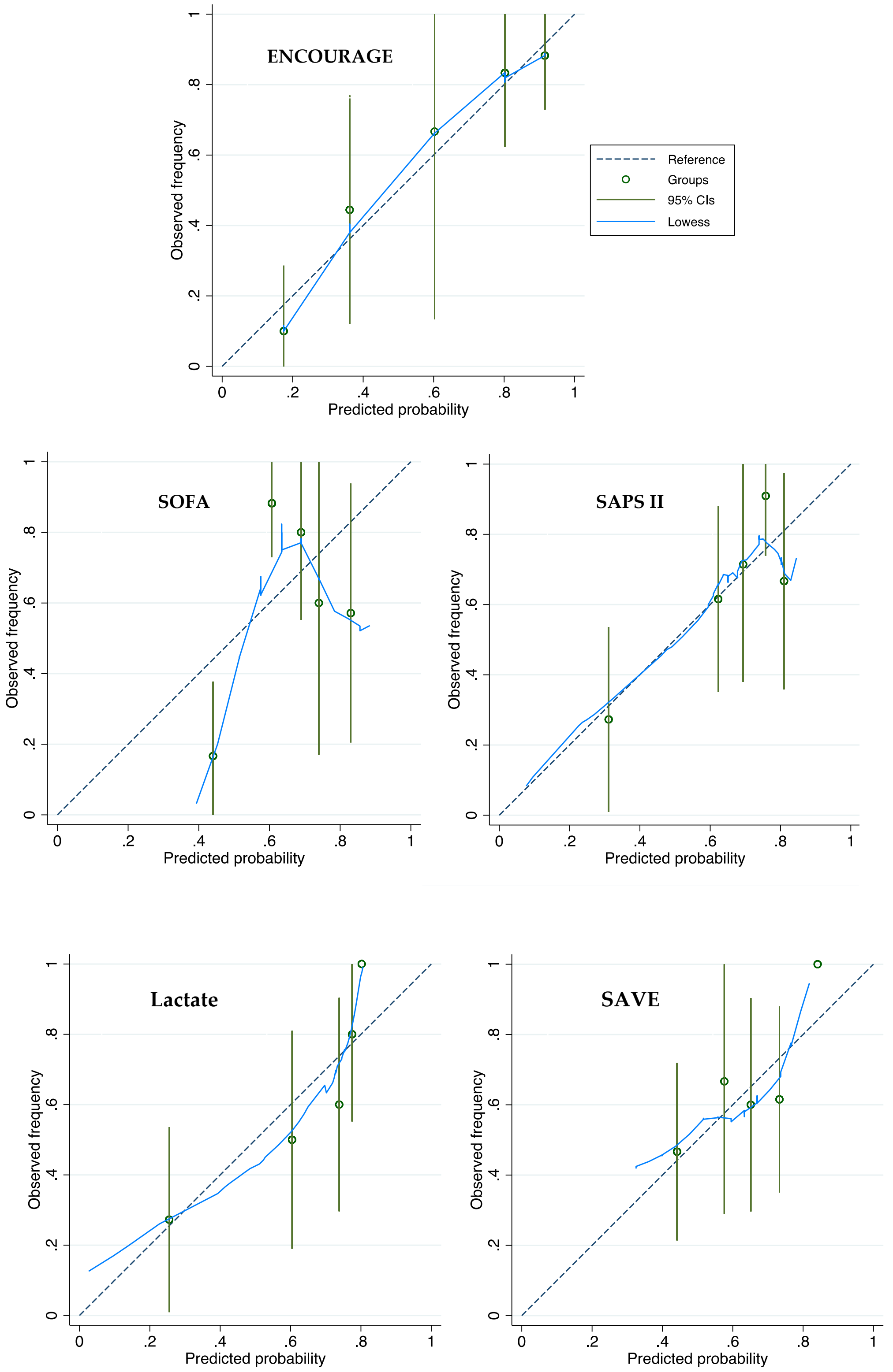

Supplement: Supplementary file 1 [file jcm-10-00956-s001.pdf]
